# A training program for obstetrics point-of-care ultrasound to 514 rural healthcare providers in Kenya

**DOI:** 10.1186/s12909-023-04886-x

**Published:** 2023-12-05

**Authors:** James Wachira, Duncan Mwangangi Matheka, Sheila Ayesa Masheti, Grace Kirigo Githemo, Sachita Shah, Matthew S. Haldeman, Mena Ramos, Kevin Bergman

**Affiliations:** 1 Global Ultrasound Institute (GUSI), San Francisco, USA; 2 Department of Obstetrics and Gynecology, School of Medicine, University of Nairobi, Nairobi, Kenya; 3 School of Health Sciences, Kenyatta University, Nairobi, Kenya; 4 Butterfly Network Inc, Massachusetts, USA; 5 Department of Emergency Medicine, University of Washington School of Medicine, Seattle, USA; 6 Department of Family and Community Medicine, University of California San Francisco, San Francisco, USA

**Keywords:** Reproductive health, Ultrasound, Education, Butterfly IQ probes, OB POCUS, Hand-held ultrasound

## Abstract

**Background:**

Ultrasound is a crucial and effective diagnostic tool in medicine. Recent advancements in technology have led to increased use of point-of-care ultrasound (POCUS). Access to ultrasound equipment and training programs in low-and middle-income countries (LMICs) is limited. Despite the World Health Organization (WHO) recommendations for universal antenatal ultrasounds, POCUS for reproductive health applications has not been widely used in LMICs. We describe here the feasibility of implementation of a training of obstetrics point-of-care ultrasound (OB POCUS) for high-risk conditions in rural public healthcare facilities in Kenya with partnership from Butterfly Network, Global Ultrasound Institute, and Kenyatta University.

**Methods:**

As part of the initiation of a large-scale implementation study of OB POCUS, clinician trainees were recruited from rural Kenyan hospitals for participation in a series of five-day POCUS workshops held between September and December 2022. Trainers provided brief didactic lessons followed by hands-on training with live models and at regional clinical sites for 5 OB POCUS applications. Instructor-observed assessment of students’ scanning and image interpretation occurred over the training period. Assessment of knowledge and confidence was performed via an online pre-test and post-test as well as Objective Structured Clinical Examination (OSCE) was administered at course completion.

**Results:**

Five hundred and fourteen mid-level Health Care Providers (HCPs) in Kenya were trained over a three-month period through in-person didactic sessions, bedside instruction, and clinical practice over a 5-day period with a trainer: trainee ratio of approximately 1:5. Out of the 514 trained HCPs, 468 were from 8 rural counties with poor maternal and neonatal outcomes, while the remaining 46 were from nearby facilities. OB POCUS topics covered included: malpresentation, multiple gestation, fetal cardiac activity, abnormalities of the placenta and amniotic fluid volume. There was marked improvement in the post training test scores compared to the pretest scores.

**Conclusion:**

Our implementation description serves as a guide for successful rapid dissemination of OB POCUS training for mid-level providers. Our experience demonstrates the feasibility of a short intensive POCUS training to rapidly establish specific POCUS skills in efforts to rapidly scale POCUS access and services. There is a widespread need for expanding access to ultrasound in pregnancy through accessible OB POCUS training programs. An implementation study is currently underway to assess the patient and systems-level impact of the training.

**Supplementary Information:**

The online version contains supplementary material available at 10.1186/s12909-023-04886-x.

## Background

Patient access to ultrasound in low- and middle-income countries (LMICs) remains a major obstacle, compromising clinician decision-making and patient outcomes [1]. Point-of-care ultrasound (POCUS) is an invaluable adjunct to bedside diagnostic evaluation [2]. POCUS refers to ultrasound performed at the patient’s bedside by the clinician to provide care using a fast, portable, and non-invasive diagnostic tool [2]. It has been shown to improve the quality and safety of patient care through reducing time to diagnosis, improving diagnostic accuracy, and maximizing procedural safety [3].

There is substantial evidence to support the clinical value of POCUS to several specialties [2–5]. For instance, POCUS has been widely used in cardiac, lung, and abdominal scanning to detect many pathologies such as pleural effusion, pneumothorax, pneumonia, interstitial syndromes [2, 3], hydronephrosis, hepatomegaly [6], splenomegaly, and ascites [6]. Limited obstetric ultrasound improved accurate identification of cases and could be beneficial in a resource-limited maternity triage setting to improve midwives' diagnoses and clinical decision-making. Surprisingly, there is limited data on the use or integration of this paradigm-shifting technology into routine obstetrical practice in LMICs [3]. This is partly due to lack of ultrasound machines combined with lack of familiarity with the tools [3]. As POCUS is a relatively new technology, most frontline physicians in LMICs have little or no experience with its use. According to Kagima et al., the lack of POCUS training for clinicians, limited resources, and a fragmented healthcare infrastructure have affected the clinician’s capability, motivation, and opportunity in performing POCUS in LMICs [2].

POCUS is highly operator-dependent [3], and must be performed by competent practitioners [7]. Safe, competent, and effective use of POCUS requires training to close gaps in learners’ knowledge and skill [3, 8]. Physicians who are proficient in its use can quickly answer specific clinical questions at the bedside [3]. Fortunately, recent data suggest that those keen to learn POCUS can obtain adequate proficiency with minimal training [3, 6, 8].

Whilst several national and international bodies have developed specialty-specific POCUS curricula, at the time of the training, there had not yet been an approved obstetrics POCUS (OB POCUS) curriculum in Kenya [7–10]. POCUS is applicable to many medical specialties, including obstetrics. The WHO recommends every woman to have at least one ultrasound scan during pregnancy [11]. In resource limited settings these is a challenge due to unavailability of ultrasound machines and the technical expertise to perform the scans. However, it has been demonstrated that with training, clinicians can comfortably conduct POCUS. Indeed, since many LMIC clinicians practice obstetrics at some level, OB POCUS is likely relevant to the majority of LMIC clinicians’ practice [12, 13]. We report our experience in training 514 HCPs across 8 counties in Kenya on obstetrics POCUS.

## Methods

Our training initiative was nested in a larger ongoing study on the impact of OB POCUS on patient outcomes, health systems, and policy and is a collaboration with three partners: (1) Global Ultrasound Institute (GUSI), which served as the training partner; (2) Butterfly Network, the ultrasound solution; and (3) Kenyatta University (KU), which served as the research and recruiting partner. Out of the 514 HCPs who were trained, 468 were from 8 rural counties with poor maternal and neonatal outcomes [14], while the remaining 46 were from KU and nearby facilities such as Kibera, Kiandutu, Gatundu, Kiambu, Thika, Ruiru, University of Nairobi, and Aga Khan University hospital (Table 1 below).

**Table 1:** showing per county number of participants trained and number of butterfly IQs received per facility

County	Total no. of facilities receiving butterfly IQs	Total no. of participants trained
Kilifi	14	41
Samburu	12	31
Nakuru	64	99
Kitui	23	63
Baringo	17	46
Taita Taveta	15	33
Kakamega	51	109
Turkana	28	46
Other (Kenyatta University Skills Lab, etc.)	-	46
Total	224	514

As part of the Butterfly probe distribution project, the Kenyan team embarked on training 514 HCPs in OB POCUS skills specifically for pregnant patients in the second and third trimesters. This entailed knowledge and skills acquisition in five key thematic areas namely: overview scan for multiple gestation, fetal presentation and lie, placental location, amniotic fluid assessment, and fetal heart rate assessment. The project aimed to train the 514 HCPs over a period of 10 weeks. The learners were trained in groups of 50 HCPs every 5 days, with 10 instructors every day. Learners were issued Butterfly iQ + ™ hand-held ultrasound probes and Apple iPads loaded with just-in time education which they used during the training and later at their local work sites.

### Participants

Kenyatta University selected 8 out of the 10 high priority counties as identified by USAID to have high maternal and neonatal morbidity and mortality rates [14]. These counties were Kitui, Kilifi, Kakamega, Nakuru, Taita Taveta, Baringo, Samburu, and Turkana. The HCPs trained included nurses, midwives, clinical officers, medical officers, radiographers, and sonographers. Most of the learners were non-physicians/non-sonographers in order to task-shift diagnostic ability to front-line providers since the majority of obstetric care is provided by them. Selected faculty members from Kenyatta University and selected HCPs from nearby health facilities in Kiambu county were also trained, with the latter facilities serving as demonstration centers for the trainings’ practical sessions.

### Instructors

OB POCUS instructors were selected from various disciplines in the medical field. These included sonographers, resident physicians in obstetrics and gynecology and family medicine, as well as an emergency physician. All had some level of ultrasound experience prior to the project.

Instructor education sessions, entitled, “Lunch and learn sessions,” were held on 3 occasions. During each, master POCUS instructors taught the program’s instructors the course content as well as effective methods in POCUS education. OB POCUS topics in the course content adhered to the International Society for Ultrasound in Obstetrics and Gynecology (ISUOG) standard ultrasound techniques [15]. In addition to reviewing the course content, instructors also had an opportunity to scan pregnant models themselves, which helped in harmonizing knowledge and passing on skills to future learners. Instructors that required more time with the Butterfly iQ + ™ probe were permitted to borrow it for a week and obtain additional practice at their clinical sites.

### Curriculum

Prior to each workshop, trainees completed a knowledge pre-test (see Additional file 1). The course itself was a structured 5-day training program employing online resources, didactic lectures, live demonstration on pregnant models, and practice opportunities with live patients in various hospitals near KU. During the training, it was emphasized that POCUS’ aim was to answer specific clinical questions to aid clinical decision-making, and appropriate indications for referral were reviewed. The training was followed by an observed assessment of scanning and diagnostic interpretation and a knowledge post-test (see Additional file 1). Upon returning to their home clinical sites, learners were offered remote image review opportunities through the GUSI online platform as well as continued support from other colleagues through WhatsApp groups.

### Lectures

The lectures were structured around 6 main themes: clinical case, normal scan findings, pathology and pitfalls, troubleshooting, clinical integration, and summary or take-home points. These were delivered as 30-min sessions, followed by a question-and-answer session for 5 min.

Following each lecture, live demonstration was then done with a ratio of 1 instructor to 5 learners. The instructor would demonstrate to the learners the expected skill to be gained and allow each of them to practice. During these hands-on sessions, the following points were also emphasized:

- Professionalism and etiquette in terms of behavior and speech towards the pregnant models.
- Participation of all learners and allowing all participants to scan without interruption or voiceovers.
- Probe care and safety of machines, including infection control.

On the first day of each training, learners were given logbooks (see Additional file 2). to record their scans throughout the week. Each learner was expected to have at least 20 scans in each of the 5 thematic areas by the end of the training period. Learners who appeared to be struggling during the skills sessions were also identified and personalized attention given to them. Learners were encouraged to rotate between different instructors in order to benefit from varying teaching styles.

Table 2 shows the 5-day training schedule.

**Table 2:** Training schedule

**Day 0**		
**Activities**	**Objective**	
• Learners received from their different counties and registered. • Issued with a Butterfly iQ + ™ probe, an iPad, and relevant accessories. • The iPads were preloaded with learning materials, pre and posttests, as well as surveys.	• Surveys were to be filled out by the learners at different time points: at month 1, 6 and 12 post training to assess the proficiency and impact of the knowledge and skills learned. • A patient survey to assess their experience and difference they felt Point of Care Ultrasound made to their management and outcome.	
**Day 1**		
**Activity 1 and objectives**	**Activity 2 and objectives**	**Activity 3 and objectives**
• **Introduction lecture and pre-recorded videos** on navigating the butterfly application, introduction to ultrasound terminology, probe movements and handling, device cleaning, charging and maintenance. • Live demonstration session objectives: • How to unlock the device, and open the butterfly application • How to save images and video clips, as well as create folders for each patient • General features of the app such as presets, modes, freeze, saving images, and how to take measurements • Essence of adequate depth and gain in achieving optimum image quality	• **Overview and fetal number.** Session taught how to perform a quick overview scan and determine the fetal number. If the fetal number was uncertain despite the overview scan, learners were urged to repeat the scan from multiple angles. • The live demonstration session objectives included: • Demonstrate and practice on how to hold the probe and probe marker orientation • Stabilization of the probe by anchoring the scan hand to the learner’s skin • Practice longitudinal and transverse probe placement • Practice probe movements to scan the whole uterus • Recognize fetal head to determine number of fetuses present • Clinical Integration: Identification of multiple gestation as a high-risk condition	• **Fetal presentation and lie.** The key message from this session included how to evaluate the relationship of the fetal head to maternal pelvis. Learners were instructed to start the scan from the maternal symphysis pubis, how to identify the maternal urinary bladder, and how to identify the fetal head. Referral of the patient was recommended for any non-cephalic presentation as delivery would likely be complicated • Live demonstration session objectives included: • Identifying the fetal presenting part and differentiating cephalic from breech presentations • Identifying the fetal spine to determine the lie (longitudinal, transverse or oblique) • Ensuring proper probe placement and orientation
**Day 2**		
**Activity 1 and objectives**	**Activity 2 and objectives**	**Activity 3 and objectives**
• **Placenta location** session focused on locating the placenta as a large hypoechoic structure adherent to the uterine wall, noting the placental tips, relating the lower tip to the cervix and fetal presenting part, and referring the patient if a low-lying placenta or previa was suspected. • The live demonstration session objectives included: • Placing probe on maternal pubic symphysis and identification of the cervix • Identifying the placenta and its location in relation to the presenting part • Determining the most distal part of the placenta and ruling out low lying placenta • Clinical Integration: Identification of low-lying and placenta previa as high-risk conditions	• **Fetal heart rate assessment** session demonstrated how to locate the fetal heart and use M-mode to measure fetal heart rate. • The live demonstration session objectives included: • Identifying the fetal heart • Optimize the image by adjusting probe position and depth • How to use M-mode to measure fetal heart rate • Clinical Integration: Identification of fetal distress	• **Amniotic fluid assessment** session instructed learners how to locate the deepest vertical pocket of amniotic fluid and evaluate volume of amniotic fluid using the single deepest pocket (SDP) method. Learners were also taught how to rule out obstructing cord using color doppler. • The live demonstration session objectives included: • How to divide the maternal abdomen into four quadrants • Identify amniotic fluid pockets free of fetal parts and umbilical cord • Use of color doppler to identify and confirm umbilical cord • Probe placement and measuring of the deepest pocket • Clinical Integration: Identification of oligo- and poly-hydramnios
**Day 3 and Day 4**		
**Activities and objectives**		
• Five public health facilities near Kenyatta University with high numbers of antenatal clients were identified, and learners were transported to these facilities for the opportunity to scan actual patients • One instructor would be assigned to supervise 4-5 learners in this setting	• Patients were explained the objectives of the scan and gave informed consent prior to any scanning • Scanning was restricted to the 5 thematic areas taught during the lectures and live demonstration. Every learner was expected to have a minimum of 20 scans throughout the week in each of the 5 thematic areas	• Of note, if any abnormal findings were discovered during the course of scanning patients, the patients’ primary medical team were notified and a formal ultrasound scan was recommended
**Day 5**		
**Activity 1**		**Activity 2**
**Assessment** • Learners that had at least 20 scans in each thematic area were allowed to sit for the exam • The exam was structured as an Observed Structured Clinical Exam (OSCE) with each learner assigned to one instructor for a period of 15 min (see Additional file 3) • Areas that were examined included probe basics, image optimization, image acquisition and interpretation of the 5 thematic areas that had been taught through the week	• Learners were deemed to have failed the assessment if they: had a score of zero in any of the 5 sections on the assessment tool, or had a total score of less than 13 out of a possible 26. If a learner failed, a remedial assessment was offered with a different instructor on either a specific section or the whole assessment • After completion of the OSCE, two post-tests, including both the Butterfly survey on the iPad and also a survey on the GUSI website, were completed by the learners. Both post tests were identical in content to the pre-tests	• **Session on final thoughts** was held where emphasis was made on probe care • Learners were also shown how to share saved images with colleagues or for consultation, as well as what to do in case their devices got lost • Learners who completed the 5-day sessions successfully received a certificate at the end of the course

### Instructors’ daily reflections

At the end of each day, the instructors brainstormed on the day's activities. They highlighted things that worked and those that did not. This proved very insightful and provided areas of improvement for the subsequent training weeks.

Table 3 highlights instructors’ daily reflections.

**Table 3:** Instructors’ daily reflections

1. Learners reminded to watch out for signs of distress in the mother and allow her to rest
2. Introduction of a form to record ‘abnormal’ pocus findings during training for communication to the primary healthcare provider
3. Preferred order of teaching and skills acquisition in the 5 thematic areas noted to be:
i. Overview and fetal number
ii. Fetal presentation and lie
iii. Placenta location
iv. Fetal heart rate assessment
v. Amniotic fluid assessment
4. Pre requisites for taking the OSCE assessment (at least 20 scans for each of the 5 thematic areas) was done during the training to encourage commitment and seriousness from the learners
5. Weak learners were identified and instructors would give them more attention and patience. Extra scanning sessions at the end of the day were also allocated to them
6. Learners encouraged to form small study groups to further enhance their knowledge
7. Instructors were encouraged to demonstrate the ultrasound skill first before letting the learners start scanning
8. Asking of questions and seeking clarifications was highly encouraged from the learners
9. Practical session on probe movements and image orientation on the screen added to help learners further grasp the concept
10. All learners encouraged to observe decorum and dress code in clinical areas during scanning sessions at the health facilities

### Follow up

Having attained knowledge and skills to conduct a second and third trimester OB POCUS, learners were expected to inculcate it in their day-to-day clinical practice at their facilities. Additionally, they were encouraged to teach their colleagues. Through GUSI, free access to their website and content was offered to learners. This allowed for:

- Refreshing of knowledge and scanning technique on the 5 thematic areas
- Assess to recorded video lectures and live demonstrations
- Uploading of scans by the learners from the devices to the GUSI online platform, with subsequent image review and feedback from GUSI instructors.

## Results

### Pre assessment and post assessment

The mean test score was 52.8% for the 446 learners that undertook the pre assessment. In comparison, the mean test score in the post assessment was 90.6% for the 432 learners that took it. This demonstrated a marked improvement in image recognition, theoretical knowledge in the 5 thematic areas and clinical application after 5 days of training.

### OSCE

Of the 489 learners that did the OSCE, 99% (486/489) attained the pass mark of 13 out of 26. The mean test score was 87.3% - demonstrating a good grasp in obstetric POCUS skills. Learners who did not attain the pass mark were allowed to retake the OSCE and passed on the second attempt.

## Discussion

Ultrasound is an extremely useful diagnostic imaging modality. It is noninvasive, provides real-time information, and is completely safe since it has no radiation exposure [10]. The use of POCUS is generally on the rise worldwide, but with challenges in LMICs partly due to lack of ultrasound machines and lack of familiarity with the tools [3]. The current paper reports the experience in training 514 HCPs in Kenya on obstetrics POCUS following a donation of hand-held probes from Gates Foundation through GUSI-Butterfly-KU partnership.

### POCUS training

The current project employed a structured 5-day training program for HCPs encompassing online resources, didactic lecture sessions, live demonstrations, practice opportunities in various hospitals, assessment and a system for remote post-course support. Although effective instruction in the short training time available remains a challenge, it can be quite effective if used optimally. Fortunately, recent data suggest that those keen to learn POCUS can obtain adequate proficiency with minimal training, including online web-based training [3, 6, 8, 9, 16].

During the training, it was emphasized that the aim of POCUS was to answer specific clinical questions that aid clinical decision-making to optimize patient outcomes [16–18]. POCUS therefore aids in making a bedside, real-time diagnosis and to enhance clinical decision-making, but not to replace the traditional or comprehensive ultrasound examinations. Referrals to sonographers and radiographers for full comprehensive scans were encouraged even for patients who had a POCUS.

### Who should be trained

The current project trained HCPs currently involved in maternal care such as nurses, midwives, clinical officers, medical officers, radiographers and sonographers. Since POCUS is inconsistently taught in medical school [8, 10], several studies have supported the incorporation of POCUS training in undergraduate medical education [3]. Studies have recommended integration of ultrasound training in undergraduate and residency training programs [19]. For instance, the majority of Canadian medical schools teaching ultrasound were able to devote 1 to 5 h per year in instruction time [6]. In addition, POCUS instruction as early as first and second year medical school has been shown to enhance knowledge acquisition and retention [6]. Surveys have shown that 50% of Canadian and 62% of US medical schools have integrated ultrasound into their curriculum, with 60% beginning in first-year [6, 8, 19]. These findings are part of a worldwide trend [9].

Jarwan et al. (2020) from Saudi Arabia assessed the needs and barriers to POCUS training among medical interns. They noted that POCUS is applicable to medical interns but significant skill gaps existed, including lack of time for training. They concluded that prioritizing the training of residents in POCUS would be a more effective use of the finite resources available for medical education instead of training medical interns who were mainly keen on medical licensing and applying for residency [3].

### Resources

This study is part of a larger funded initiative to improve diagnostic imaging access in order to propel Kenya towards achieving improvements in their maternal health outcomes. To meet the WHO recommendations for antenatal imaging, health departments should prioritize the acquisition of hand-held ultrasound machines paired with clinician training, which may serve to reduce patient barriers to necessary medical imaging. Making bedside imaging available to critically ill patients and those that require imaging outside of normal business hours will allow clinicians to make life-saving diagnoses of high-risk conditions in their most critical patients [2, 3]. The integration of POCUS into routine clinical practice requires a substantial up-front investment, and the resources available for medical education are generally limited in LMICs. Further research should examine cost-benefit analysis, improvements and effects of routine OB POCUS on referral patterns and impact on patient health outcomes.

### Limitations

The study has potential limitations. The assessment multiple choice questions had not been standardized nor validated in any other study or setting. They were developed by the authors and therefore may require reframing and validation in other training for a more widespread application.

## Conclusions

We report the experience of training 514 HCPs in obstetric POCUS using didactic lectures and hands-on experience over a 5-day period using the ISUOG standard ultrasound techniques. Follow up data will be collected to assess the impact of the current training and deployment of portable ultrasound devices to mid-level HCPs in hard-to-reach areas with little or no access to ultrasound services.

Larger, more widespread OB POCUS training programs and estimation of their clinical impact are needed in LMIs. More broadly, the development of widespread POCUS training curricula for a wide variety of clinical applications should be embedded as an integral component of medical education in these settings. We encourage the integration of ultrasound training in medical schools throughout LMICs, which could have enormous potential impact on patient care in the future.

### Supplementary Information

**Additional file 1.** Pre and Post Test. Pre and Post Test used to assess baseline knowledge and gained knowledge of learners.

**Additional file 2.** Logbook for Basic Obstetric Ultrasound. Learner’s logbook for tracking and recording ultrasound scans completed during the training.

**Additional file 3.** OSCE Evaluation Form. Observed Structured Clinical Exam (OSCE) used to evaluate learners on the final day of training.

## Data Availability

The data and materials supporting the conclusions of this article are included within the article and its additional files.

## Abbreviations

GUSI: Global Ultrasound Institute
HCPs: Health Care Providers
ISUOG: International Society of Ultrasound in Obstetrics and Gynecology
KU: Kenyatta University
LMICs: Low- and Middle-Income Countries
OB POCUS: Obstetrics Point of Care Ultrasound
OSCE: Objective Structured Clinical Examination
POCUS: Point of Care Ultrasound
SDP: Single Deepest Pocket
US: United States
