# Supplementary material for: A training program for obstetrics point-of-care ultrasound to 514 rural healthcare providers in Kenya
Source: BMC Med Educ. 2023 Dec 5;23:922. doi: 10.1186/s12909-023-04886-x (PMC10698920; doi:10.1186/s12909-023-04886-x)
Supplement: Supplementary file 1 — Additional file 1. Pre and Post Test. Pre and Post Test used to assess baseline knowledge and gained knowledge of learners. [file 12909_2023_4886_MOESM1_ESM.docx]

**Additional File 1**

- File name: Additional file 1
- File format: Ms Word .docx
- Title of data: Pre and Post Test
- Description of data: Pre and Post Test used to assess baseline knowledge and gained knowledge of learners.

**Kenya Obstetrics Pre and Post-Course Test**


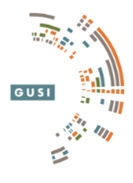


**Global Ultrasound Institute**

Your 20 year old patient presents to you at 30 weeks gestation because she is not feeling her baby move. You perform a physical exam and do an ultrasound. The baby’s fetal heart rate was measured at 90 beats per minute. What ultrasound mode was used to evaluate fetal heart rate?

· 2 Dimensional (2D) Mode

· Brightness (B) Mode

· Color Doppler Mode

· Power Doppler Mode

· Motion (M) Mode

A 23 year old G2P1 at 32 weeks gestation is presenting with vaginal bleeding. You perform an ultrasound above the pubic symphysis and see the following image? What letter in the image below is suggestive of Placenta Previa?

· A

· B

· C

· D

· E

A 30 year old G3P1 at 35 weeks gestation by last menstrual period is being evaluated for the first time by you. You perform the first ultrasound of her pregnancy. You decide to do an overview exam and see the following:

This finding highlighted in yellow suggests which of the following?

Singleton Pregnancy

Membrane suggesting more than 1 fetal sac

Oligohydramnios

Polyhydramnios

True or False. The single deepest pocket measurement (SDP) is less accurate than amniotic fluid index (AFI) for amniotic fluid assessment. The SDP leads to more unnecessary labor induction than AFI.

· True

· False

Your patient at 34 weeks gestation has a fundal height of 28cm. You perform an ultrasound and find the single deepest pocket to be less than 1.2cm. What is your diagnosis?

· Oligohydramnios

· Polyhydramnios

· Placenta Previa

· Low Lying Placenta

· Breech Presentation

What best describes fetal presentation?

· The axis the fetal spine relative to the maternal spine

· The axis the fetal spine relative to the maternal pelvis

· The part of the fetus that is overlying the pelvis

· The part of the fetus that is overlying the maternal umbilicus

What is structure is visible in blue and red?

· Umbilical Cord

· Fetal Heart

· Placenta

· Cervix

· Fetal Head

What best describes fetal lie?

· The axis of the fetus relative to the maternal spine

· The axis of the fetus relative to the maternal pelvis

· The part of the fetus that is overlying the maternal pelvis

· The axis of the fetus relative to the umbilical cord

PRE-TEST

1. You are performing an amniotic fluid assessment by measuring the single deepest pocket and find it to be within normal range at 4.5cm. What best describes the sonographic appearance of amniotic fluid?

Anechoic

Hyperechoic

Heterogeneous

Isoechoic

Reverberation

2. Your 20 year old patient presents to you at 30 weeks gestation because she is not feeling her baby move. You perform a physical exam and do an ultrasound. What best describes the baby’s fetal heart rate?

Fetal Bradycardia

Fetal Tachycardia

Normal Fetal Heart Rate

3. Your 20 year old patient presents to you at 30 weeks gestation because she is not feeling her baby move. You perform a physical exam and do an ultrasound. The baby’s fetal heart rate was measured at 90 beats per minute. What ultrasound mode was used to evaluate fetal heart rate?


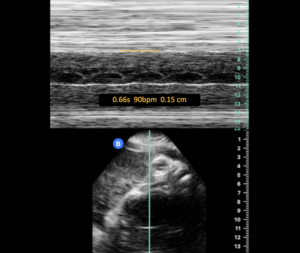

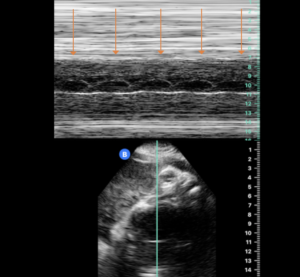


2 Dimensional (2D) Mode

Brightness (B) Mode

Color Doppler Mode

Power Doppler Mode

Motion (M) Mode

A 23 year old G2P1 at 32 weeks gestation is presenting with vaginal bleeding. You perform an ultrasound above the pubic symphysis and see the following image? What letter in the image below is suggestive of Placenta Previa?


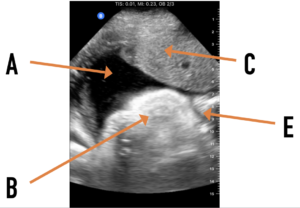


A

B

C

D

E

A 30 year old G3P1 at 35 weeks gestation by last menstrual period is being evaluated for the first time by you. You perform the first ultrasound of her pregnancy. You decide to do an overview exam and see the following:


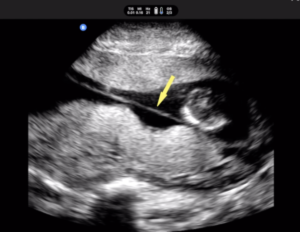


6. This finding highlighted in yellow suggests which of the following?

Singleton Pregnancy

Membrane suggesting more than 1 fetal sac

Oligohydramnios

Polyhydramnios

7. True or False. The single deepest pocket measurement (SDP) is less accurate than amniotic fluid index (AFI) for amniotic fluid assessment. The SDP leads to more unnecessary labor induction than AFI.

True

False

8. Your patient at 34 weeks gestation has a fundal height of 28cm. You perform an ultrasound and find the single deepest pocket to be less than 1.2cm. What is your diagnosis?


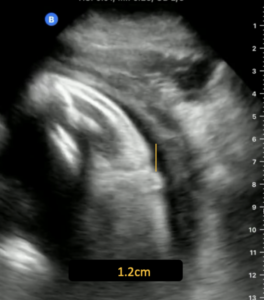


Oligohydramnios

Polyhydramnios

Placenta Previa

Low Lying Placenta

Breech Presentation

9. What best describes fetal presentation?

The axis the fetal spine relative to the maternal spine

The axis the fetal spine relative to the maternal pelvis

The part of the fetus that is overlying the pelvis

The part of the fetus that is overlying the maternal umbilicus

10. What is structure is visible in blue and red?


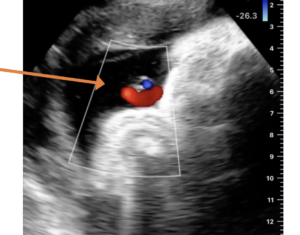


Umbilical Cord

Fetal Heart

Placenta

Cervix

Fetal Head

11. What best describes fetal lie?

The axis of the fetus relative to the maternal spine

The axis of the fetus relative to the maternal pelvis

The part of the fetus that is overlying the maternal pelvis

The axis of the fetus relative to the umbilical cord
