# Supplementary material for: A training program for obstetrics point-of-care ultrasound to 514 rural healthcare providers in Kenya
Source: BMC Med Educ. 2023 Dec 5;23:922. doi: 10.1186/s12909-023-04886-x (PMC10698920; doi:10.1186/s12909-023-04886-x)
Supplement: Supplementary file 2 — Additional file 2. Logbook for Basic Obstetric Ultrasound. Learner’s logbook for tracking and recording ultrasound scans completed during the training. [file 12909_2023_4886_MOESM2_ESM.docx]

**Additional File 2**

- File name: Additional file 2
- File format: Ms Word .docx
- Title of data: Logbook for Basic Obstetric Ultrasound
- Description of data: Learner’s logbook for tracking and recording ultrasound scans completed during the training.

**Logbook for Basic Obstetric Ultrasound**


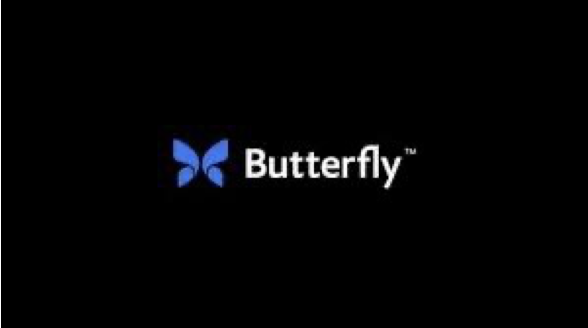

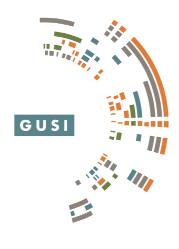


Global Ultrasound Institute

and

The Butterfly Network

Name: ________________________________________

Occupation: ____________________________________

Home Facility: __________________________________

Home County: __________________________________

Minimum scan requirements:

- 20 Overview/Fetal number scans
- 20 Fetal presentation scans
- 20 Fetal heart rate using M-mode scans
- 20 Amniotic fluid assessment scans using Single Deepest Pocket (SDP)
- 20 Placental location scans

|  | Date | Clinical  Site | Overview/Fetal Number  (Indicate if singleton or multiple) | Fetal  presentation(indicate if cephalic or Breech ) | Fetal Heart Rate-(M-mode)  (Indicate if normal, Bradycardia or Tachycardia)  (Normal 110-160bpm) | Amniotic Fluid (SDP)  (Indicate value and if normal or otherwise) | Placenta Location  (Indicate if Anterior,Posterior,Fundal or Previa) | Findings  (Comment on any abnormal findings if any,if not indicate Normal.) |
| --- | --- | --- | --- | --- | --- | --- | --- | --- |
|  |  |  | *Supervisor to place initials in these columns as scans are completed.* | | | | |  |
| 1 |  |  |  |  |  |  |  |  |
| 2 |  |  |  |  |  |  |  |  |
| 3 |  |  |  |  |  |  |  |  |
| 4 |  |  |  |  |  |  |  |  |
| 5 |  |  |  |  |  |  |  |  |
| 6 |  |  |  |  |  |  |  |  |
| 7 |  |  |  |  |  |  |  |  |
| 8 |  |  |  |  |  |  |  |  |
| 9 |  |  |  |  |  |  |  |  |
| 10 |  |  |  |  |  |  |  |  |
| 11 |  |  |  |  |  |  |  |  |
| 12 |  |  |  |  |  |  |  |  |
| 13 |  |  |  |  |  |  |  |  |
| 14 |  |  |  |  |  |  |  |  |
| 15 |  |  |  |  |  |  |  |  |
| 16 |  |  |  |  |  |  |  |  |
| 17 |  |  |  |  |  |  |  |  |
| 18 |  |  |  |  |  |  |  |  |
| 19 |  |  |  |  |  |  |  |  |
| 20 |  |  |  |  |  |  |  |  |
| TOTALS: | | |  |  |  |  |  |  |
