# Supplementary material for: A training program for obstetrics point-of-care ultrasound to 514 rural healthcare providers in Kenya
Source: BMC Med Educ. 2023 Dec 5;23:922. doi: 10.1186/s12909-023-04886-x (PMC10698920; doi:10.1186/s12909-023-04886-x)
Supplement: Supplementary file 3 — Additional file 3. OSCE Evaluation Form. Observed Structured Clinical Exam (OSCE) used to evaluate learners on the final day of training. [file 12909_2023_4886_MOESM3_ESM.docx]

**Additional File 3**

- File name: Additional file 3
- File format: Ms Word .docx
- Title of data: OSCE Evaluation Form
- Description of data: Observed Structured Clinical Exam (OSCE) used to evaluate learners on the final day of training.


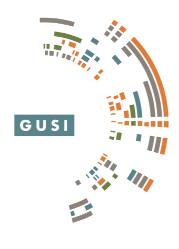
**OSCE Evaluation Form**

Global Ultrasound Institute

Kenya Obstetric Point-of-Care Ultrasound

OSCE Evaluation Form

*Instructor directions: First, review participants’ logbooks and write the total number of completed scans. Ask the participant to perform the following functions. Observe the participant and grade him/her by placing an “x” in the corresponding column for 0, 1, or 2 points. Do not assist the participant during the assessment itself.*

*Following each assessment scan, give the participant constructive feedback to help him/her master the skill.*

Name of Participant: _____________________________________ Date: ___________________

Name of Instructor: _________________________________________________

| Question | Mastered (2 points) | Proficient (1 point) | Unsatisfactory (0 points) | Comments |
| --- | --- | --- | --- | --- |
| *Image Optimization* |  |  |  |  |
| Chooses correct preset |  |  |  |  |
| Optimizes image depth |  |  |  |  |
| Optimizes image gain |  |  |  |  |
|  |  |  |  |  |
| *Overview Scan/Fetal Number* |  |  |  |  |
| Correctly performs Overview scan |  |  |  |  |
| Accurately determines correct fetal number |  |  |  |  |
|  |  |  |  |  |
| *Fetal Presentation* |  |  |  |  |
| Obtains and identifies fetal head correctly |  |  |  |  |
| Accurately interprets fetal presentation |  |  |  |  |
|  |  |  |  |  |
| *Fetal Heart Rate* |  |  |  |  |
| Obtains image of fetal heart (centered on screen, zoomed in) |  |  |  |  |
| Accurately measures FHR using M-mode |  |  |  |  |
|  |  |  |  |  |
| *Placental Location* |  |  |  |  |
| Correctly locates and identifies placenta |  |  |  |  |
| Accurately determines presence/absence of placenta previa/low-lying placenta |  |  |  |  |
|  |  |  |  |  |
| *Amniotic Fluid Assessment using SDP Method* |  |  |  |  |
| Correctly locates single deepest pocket with probe held vertically |  |  |  |  |
| Accurately measures SDP without any obstructing structures |  |  |  |  |
| *Total of each column:* |  |  | Final comments: | |
| *Final score:* |  |  |  |  |
| *Out of possible:* | 26 |  |  |  |
| *Grade: Pass/Fail* |  |  |  |  |
| *Total # completed scans from logbook* |  |  |  |  |
